# Fisher Information of Free-Electron Landau States

**DOI:** 10.3390/e23030268

**Published:** 2021-02-25

**Authors:** Takuya Yamano

**Affiliations:** Faculty of Science, Kanagawa University, 2946, 6-233 Tsuchiya, Hiratsuka 259-1293, Japan; yamano@amy.hi-ho.ne.jp

**Keywords:** Landau states, Fisher information, relative Fisher information, radial wavefunction, generalized Laguerre polynomials

## Abstract

An electron in a constant magnetic field has energy levels, known as the Landau levels. One can obtain the corresponding radial wavefunction of free-electron Landau states in cylindrical polar coordinates. However, this system has not been explored so far in terms of an information-theoretical viewpoint. Here, we focus on Fisher information associated with these Landau states specified by the two quantum numbers. Fisher information provides a useful measure of the electronic structure in quantum systems, such as hydrogen-like atoms and under some potentials. By numerically evaluating the generalized Laguerre polynomials in the radial densities, we report that Fisher information increases linearly with the principal quantum number that specifies energy levels, but decreases monotonically with the azimuthal quantum number *m*. We also present relative Fisher information of the Landau states against the reference density with m=0, which is proportional to the principal quantum number. We compare it with the case when the lowest Landau level state is set as the reference.

## 1. Introduction

The motion of electrons in a uniform magnetic field is a fundamental process in a variety of science. Specifically, the Landau states and the associated levels [1,2,3] are the most elementary quantum manifestations underlying diverse effects in condensed-matter physics, such as the De Haas van Alphen effects [4], Shubnikov de Haas effects [5], and fractional/integer quantum Hall effects [6]. Therefore, Landau states and their energy levels have attracted enormous attention concerning oscillatory behaviors observed in various electronic properties. These properties should be attributed to the forms of the underlying wavefunctions. The shape of the wavefunctions or the electric density functions has a piece of inherent information. However, research interests so far have been focused on the occupation numbers of Landau energy levels in condensed-matter systems instead of information content contained in the spatial distributions of electrons represented by the wavefunctions. Consequently, we ask a natural question: How much information do the Landau states contain, or how do we compare the information content between the different states specified by the quantum numbers?

In a different discipline, by contrast, an information-theoretical approach to atomic systems aimed at interpreting and characterizing the orbital structure has attracted much attention [7]. Among several information measures, many authors have intensively studied Fisher information [8,9] that reflects gradient content (i.e., sharpness) of the density distribution for both relativistic and non-relativistic hydrogenic atoms (e.g., [10,11,12,13]; these are only a few that are of close relevance to the present author, since the literature is vast). This preference of the information measure is because it is more sensitive in understanding the degree of localization of an electron than using Shannon entropy. Additionally, for heavier atoms, the amount of Fisher information has been explored (e.g., [14]; this is one example among many, and see references cited therein). These developments motivate us to study information measures of other quantum systems if the associated wavefunctions are available. Examples are systems under Morse, Pöschl–Teller potentials, and the quantum harmonic oscillator systems [15]. In addition to the above practical merits in atomic and molecular systems, Fisher information is at the root of various theoretical descriptions through profound connections with the foundations of quantum mechanics [16]. This connection also has wider implications for other branches in physics. For instance, the Schrödinger equation is derived from the constrained Fisher-optimization scheme and led to a new approach to non-equilibrium processes, such as the propagation of sound waves in a dilute gas [17].

Although the information quantity itself has not been a major concern in the literature of solid-state physics, some recent works relate the information concept and the quantum number *n* that specifies the Landau levels under fixed electric and magnetic fields [18,19,20]. The aim of these studies is to propose an indicator of topological phase transitions in two-dimensional topological insulators, and the behavior of Fisher information and the Rényi–Fisher entropy product have been studied for eigenstates in 2D gapped Dirac materials for several values of *n*. Contrary to the Landau states in this study, the eigenstate wavefunctions have no dependence on the azimuthal quantum number. We focus on an information-theoretical treatment for the *free-electron* Landau states; that is, we consider *free* electrons in a uniform magnetic field that also exhibit the Landau levels [21], and the analytical form of the wavefunctions are available in this setting [22]. We demonstrate the dependence of the Fisher information of the free-electron Landau states on the principal quantum number and the azimuthal quantum number. We also show that relative Fisher information against the lowest Landau state is a quantifier of dissimilarity among the states. In the next section, we provide the density function of the Landau states and show the behavior of the Fisher information. In Section 3, we analytically derive the relative Fisher information against a state with zero azimuthal quantum numbers. We summarize this study and add a discussion in the last section. The Appendix A is given for a detailed calculation of the relative Fisher information when the reference state is set as the lowest Landau state.

## 2. Fisher Information of Landau States

In this section, we first summarize the system under consideration. Landau studied the motion of a charged particle in a constant uniform magnetic field [2]. One can derive energy discretization (Landau levels) perpendicular to the magnetic field in a Cartesian coordinate system. However, the cylindrical polar coordinates are more convenient in solving Schrödinger equations to find the wave functions for systems in a magnetic field. Indeed, Landau & Lifshitz [22] uses this coordinate system. Let ρ, ϕ, and *z* specify the position of an electron with mass *M* and charge e=−|e| in cylindrical polar coordinates under the uniform magnetic field *H* added in *z*-direction. When we choose the Landau gauge for the vector potential A as (0,Hρ/2,0), the Hamiltonian operator corresponding to the Schrödinger equation ${\left(2M\right)}^{-1}{\left(\hat{p}-\left(e/c\right)A\right)}^{2}\psi =E\psi$ with $\hat{p}$ being the momentum operator is

(1) $$-\frac{\hbar^{2}}{2M}\left[\frac{1}{\rho }\frac{\partial }{\partial \rho }\left(\rho \frac{\partial }{\partial \rho }\right)+\frac{1}{\rho^{2}}\frac{\partial^{2}}{\partial \phi^{2}}+\frac{\partial^{2}}{\partial z^{2}}-\frac{1}{2}i\hbar \omega \frac{\partial }{\partial \phi }+\frac{1}{8}M\omega^{2}\rho^{2}\right]$$

where ω denotes the cyclotron frequency. The Hamiltonian does not contain the coordinate *z* (also *x*) explicitly, and the *z*-component of the momentum $p_{z}$ commutes with the Hamiltonian. Thus, $p_{z}$ is conserved, and the system has definite angular momentum in the direction of the magnetic field. By seeking the solution of the form

(2) $$\psi =\frac{1}{\sqrt{2\pi }}R\left(\rho \right)e^{im\phi }e^{i\frac{p_{z}z}{\hbar }}$$

the radial wave functions R(ρ) are expressed as (§112 in Ref. [22]):

(3) $$R_{n_{\rho },m}\left(\rho \right)=N_{n_{\rho },m}\exp\left(-\frac{\rho^{2}}{4a_{H}^{2}}\right)\rho^{\left|m\right|}F(-n_{\rho },|m|+1,\frac{\rho^{2}}{2a_{H}^{2}}),$$

where $a_{H}=\sqrt{\frac{\hbar c}{|e|H}}$ is the magnetic length parameter, and $n_{\rho }$ is the radial quantum number being a non-negative integer. The quantum number m=0,±1,±2,… is associated with the azimuthal wave function $e^{im\phi }/\sqrt{2\pi }$. The confluent hypergeometric function *F* is defined with the generalized Laguerre polynomial $L_{n}^{\left(\alpha \right)}\left(x\right)$ as

(4) $$\begin{array}{c} F\left(-n,\alpha +1,x\right)=\frac{n!\Gamma (\alpha +1)}{\Gamma (n+\alpha +1)}L_{n}^{\left(\alpha \right)}\left(x\right). \end{array}$$

The normalization condition

(5) $$\int_{0}^{\infty }R_{n_{\rho },m}^{2}\left(\rho \right)\rho d\rho =1$$

determines the factor $N_{n_{\rho },m}$ as

(6) $$N_{n_{\rho },m}=\frac{1}{a_{H}^{|m|+1}\left|m\right|!}{\left(\frac{(|m|+n_{\rho })!}{2^{\left|m\right|}n_{\rho }!}\right)}^{\frac{1}{2}}.$$

**Figure 1:**
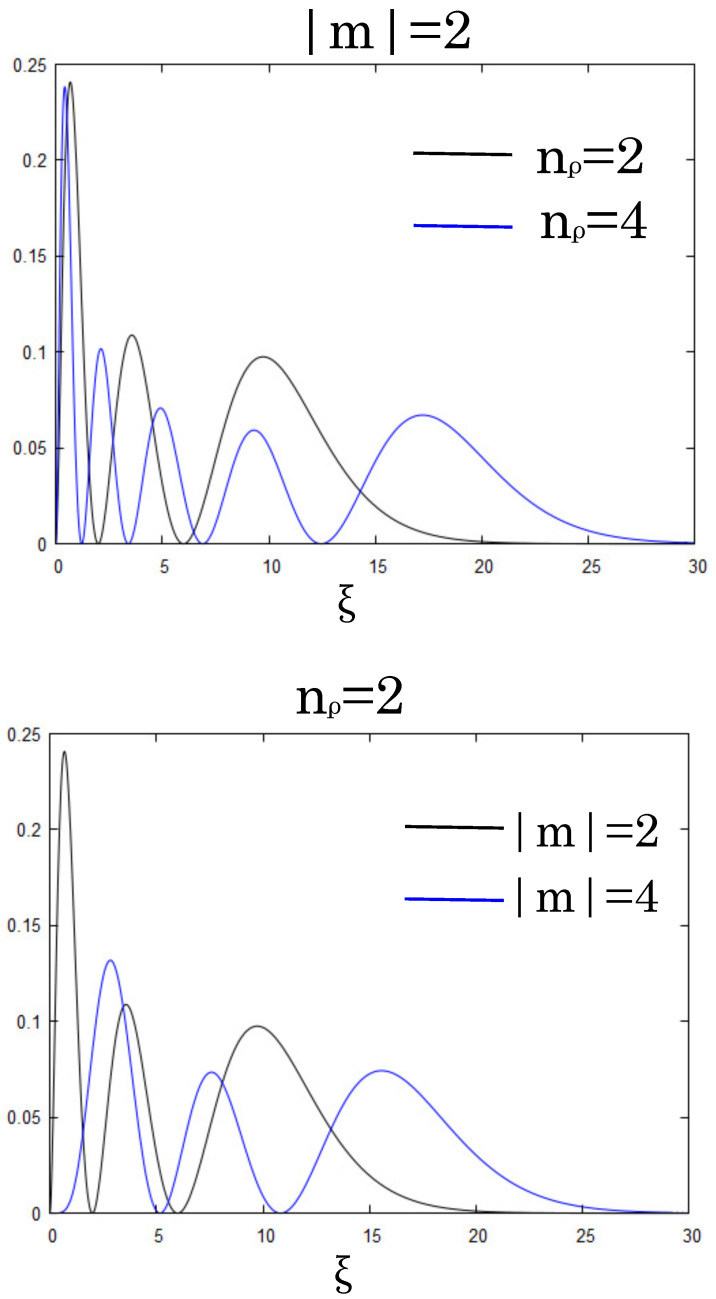
Radial probability densities of the Landau state with $n_{\rho }=2$ (black) and $n_{\rho }=4$ (blue) for the fixed quantum number |m|=2 (**upper panel**). The same with |m|=2 (black) and |m|=4 (blue) for the fixed quantum number $n_{\rho }=2$ (**lower panel**).

Changing the variable as $\xi =\rho^{2}/2a_{H}^{2}$ allows one to express the radial probability density $\eta_{n_{\rho },m}\left(\xi \right)$ of finding the particle in the states with the quantum numbers $n_{\rho }$ and *m*:

(7) $$\eta_{n_{\rho },m}\left(\xi \right)=M_{n_{\rho },m}\xi^{\left|m\right|}e^{-\xi }[F(-n_{\rho },|m|+{1,\xi )]}^{2},\;M_{n_{\rho },m}=\frac{(|m|+n_{\rho })!}{{\left(|m|!\right)}^{2}n_{\rho }!},$$

so that one assures the normalization of the density $\int_{0}^{\infty }\eta_{n_{\rho },m}\left(\xi \right)d\xi =1$. The dependency of the radial density on the magnetic field *H* enters through the new variable ξ, which is proportional to the magnetic field. The probability density of the lowest Landau state corresponds to the quantum numbers $n_{\rho }=m=0$. To grasp the dependencies on the quantum numbers, we show the behavior for some of the radial probability densities in Figure 1.

The radial density function broadens and has a more nodal structure for the higher quantum numbers (blue curves). To reflect this gradient feature of densities into an information measure, Fisher information can be appropriate one. Fisher information was originally regarded as a quality metric of the estimation procedure, and it is a function of the parameter θ in the probability density function [8]. In the particular case of one variable *x*, it is defined by $\int {\left[p_{\theta }\left(x,\theta \right)\right]}^{2}/p\left(x,\theta \right)dx$ in a valid range of *x*. However, when the probability obeys a property of shift (translation) invariance, that is, p(x|θ)=p(x−θ), it no longer depends upon θ and is equivalent to the integral of ${\left[p^{\prime }\left(x\right)\right]}^{2}/p\left(x\right)$ [16]. This shift invariance means that the variable *x* is independent of the value of the parameter θ. In this study, and in many papers as well in the literature, we refer this quantity as Fisher information [16]. Thus, the Fisher information (FI) of the radial probability density $\eta_{n_{\rho },m}\left(\xi \right)$ is defined as

(8) $$FI\left(\eta_{n_{\rho },m}\right)=\int_{0}^{\infty }\frac{1}{\eta_{n_{\rho },m}\left(\xi \right)}{\left(\frac{d\eta_{n_{\rho },m}\left(\xi \right)}{d\xi }\right)}^{2}d\xi =4\int_{0}^{\infty }{\left(\frac{d\sqrt{\eta_{n_{\rho },m}\left(\xi \right)}}{d\xi }\right)}^{2}d\xi .$$

**Figure 2:**
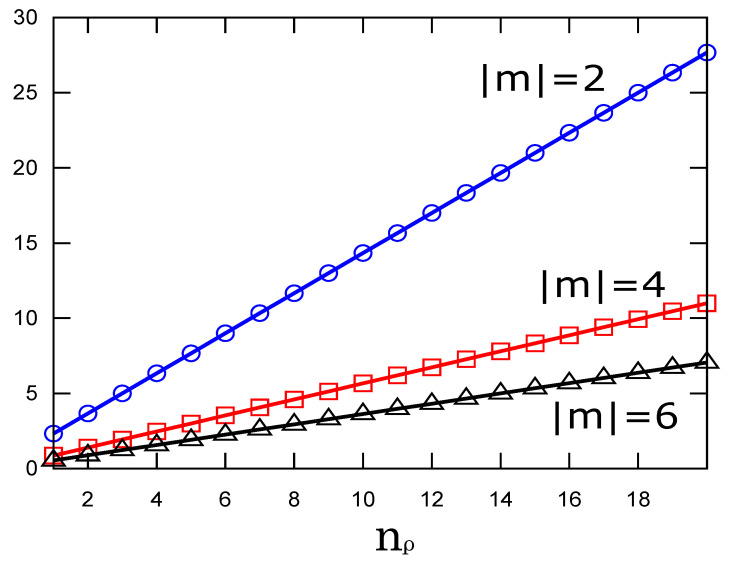
Fisher information of the radial probability density of the Landau states as a function of the radial quantum number $n_{\rho }$ for three values of |m|.

**Figure 3:**
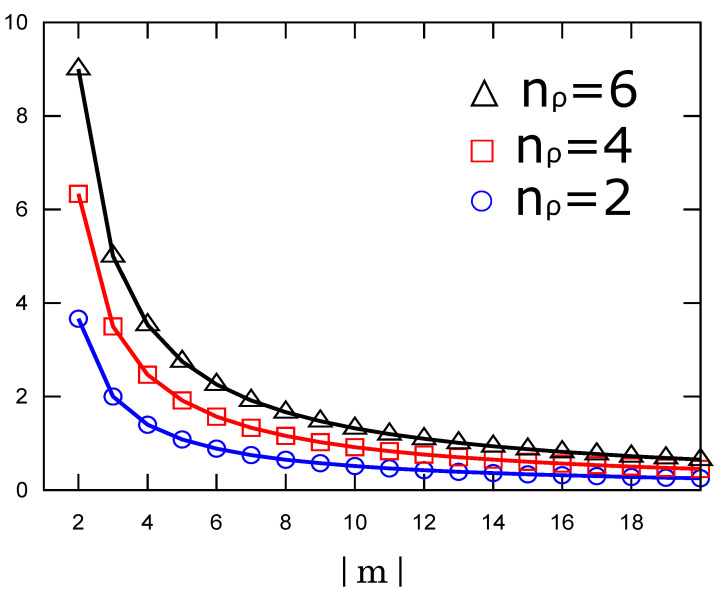
Fisher information of the radial probability density of the Landau states as a function of the azimuthal quantum number |m| for three values of $n_{\rho }$.

We use the second expression for the ease of computation. In Figure 2, we show the numerically determined values of Equation (8) as a function of the quantum number $n_{\rho }$. We observe that the information content grows linearly as $n_{\rho }$ gets large, and the slope becomes smaller as |m| becomes large. We note that the higher values of Fisher information mean a stronger localization of the probability density. On the other hand, Figure 3 shows the decreasing behaviors of Fisher information as a function of the azimuthal quantum number |m|. It indicates that states with larger radial quantum numbers have larger information for the same azimuthal quantum number.

## 3. Relative Fisher Information of Landau States

Next, we study the relative Fisher information to see how the excited states differ from a reference state. The relative Fisher information can be a suitable measure of similarity or dissimilarity between two states when one uses Fisher information contained in systems. It is recently applied to study atomic shell structures [23], atomic ionization processes and isoelectronic series [24], sinusoidal and gamma-like densities [25], one-particle densities of (non-)relativistic hydrogenic systems [12,26], the Morse potential and isotropic quantum harmonic oscillators [27,28], diatomic molecules with the pseudoharmonic potential [27], the deviation of the Pauli and Weizsäcker kinetic energy densities from the local density approximation [29], and the derivation of the Euler equation of the orbital-free excited-state density functional theory as a variational problem [30].

To use relative information, one must first choose the reference state. Here, we measure the relative information between the excited Landau level specified by $n_{\rho }$ and the lowest Landau level $n_{\rho }=0$ having the common |m|. This choice is natural, since we have set the ground state of a system as a reference state in the studies of the radial wavefunctions of hydrogen-like atoms in position space [12], quantum harmonic oscillators [27,28], and under some central potentials in both position and momentum spaces [27]. Note that the lowest Landau level wavefunction is widely used in the literature of the quantum Hall effect and various material sciences. Thus, it is expressed as

(9) $$\begin{array}{ccc} \mathrm{RFI}(\eta_{n_{\rho },m}\left(\xi \right):\eta_{0,m}\left(\xi \right)) & := & \int_{0}^{\infty }\eta_{n_{\rho },m}\left(\xi \right)|\frac{d}{d\xi }\log\left(\frac{\eta_{n_{\rho },m}\left(\xi \right)}{\eta_{0,m}\left(\xi \right)}\right){|}^{2}d\xi \\ & = & \int_{0}^{\infty }\eta_{n_{\rho },m}\left(\xi \right){\left(\frac{\eta_{n_{\rho },m}^{\prime }\left(\xi \right)}{\eta_{n_{\rho },m}\left(\xi \right)}-\frac{\eta_{0,m}^{\prime }\left(\xi \right)}{\eta_{0,m}\left(\xi \right)}\right)}^{2}d\xi . \end{array}$$

We note that $L_{0}^{\left(\alpha \right)}\left(\xi \right)=1$, thus F(0,α+1,ξ)=1. Therefore, from Equation (7), the probability density for the lowest Landau state is $\eta_{0,m}\left(\xi \right)=\xi^{\left|m\right|}e^{-\xi }/\left|m\right|!$. Substituting the probability density Equation (7) into the above definition leads to the calculation of the integral

(10) $$\begin{array}{c} 4M_{n_{\rho },m}\int_{0}^{\infty }\xi^{\left|m\right|}e^{-\xi }[F^{\prime }(-n_{\rho },|m|+{1,\xi )]}^{2}d\xi . \end{array}$$

Noting that the derivative of the generalized Laguerre polynomial with respect to ξ satisfies the relation ${\left[L_{n}^{\left(\alpha \right)}\left(\xi \right)\right]}^{\prime }=-L_{n-1}^{\left(\alpha +1\right)}\left(\xi \right)$. Thus, from Equation (4), the derivative of the confluent hypergeometric function is given as

(11) $$\begin{array}{c} F^{\prime }(-n_{\rho },|m|+1,\xi )=-\frac{\Gamma \left(|m|+1\right)n_{\rho }!}{\Gamma (n_{\rho }+|m|+1)}L_{n_{\rho }-1}^{\left(|m|+1\right)}\left(\xi \right). \end{array}$$

Invoking the orthogonality relation for the generalized Laguerre polynomial (p. 1012 in Ref. [31])

(12) $$\int_{0}^{\infty }\xi^{\alpha }e^{-\xi }L_{n}^{\left(\alpha \right)}\left(\xi \right)L_{m}^{\left(\alpha \right)}\left(\xi \right)d\xi =\frac{\Gamma (\alpha +n+1)}{n!}\delta_{m,n},$$

we find that the relative Fisher information is independent of the azimuthal quantum number |m| and is proportional to the principal quantum number:

(13) $$\begin{array}{c} \mathrm{RFI}\left(\eta_{n_{\rho },m}\left(\xi \right):\eta_{0,m}\left(\xi \right)\right)=4n_{\rho }. \end{array}$$

The absence of |m| in the measure is plausible because one compares two densities with the common *m*.

## 4. Summary and Discussion

We have revealed some striking features of Fisher information and the relative Fisher information on the radial probability density of the free-electron Landau states. First, the Fisher information increases linearly with the principal quantum number, whereas it monotonically decreases with the azimuthal quantum number. Second, relative Fisher information changes linearly in the principal quantum number when one adopts the states with m=0 as the reference probability density. In this study, we have regarded $\eta_{n_{\rho },m}\left(\xi \right)$ as the radial probability density with the rescaled variable proportional to the radial coordinate, that is, $\xi =\rho^{2}/2a_{H}^{2}$. A natural alternative that finds the electron in the range [ρ,ρ+dρ] is to use $R_{n_{\rho },m}{\left(\rho \right)}^{2}\rho$. In this case, Fisher information is determined in units of $a_{H}^{3}$. More specifically, it is $2\sqrt{2}M_{n_{\rho },m}I/a_{H}^{3}$, where *I* is the associated integral. We have confirmed that the behavior of the Fisher information as a function of $n_{\rho }$ is essentially the same as Figure 2; that is, it linearly grows with $n_{\rho }$, except that the dependence of *m* is slight compared to the separated straight lines in this figure. Therefore, to compare the difference in spreading of the radial eigenfunctions, the use of the probability density $\eta_{n_{\rho },m}\left(\xi \right)$ is fully valid as a descriptive indicator.

These findings give new insights into the fundamental properties of the Landau states. The choice of the reference probability density for the relative Fisher information is at our disposal. Another possible one is the state with m=0, that is, $\eta_{n_{\rho },0}\left(\xi \right)=e^{-\xi }{\left[L_{n_{\rho }}\left(\xi \right)\right]}^{2}$, where $L_{n_{\rho }}\left(\xi \right)=L_{n_{\rho }}^{\left(0\right)}\left(\xi \right)$ is the Laguerre polynomial. In this case, one must evaluate the integral

(14) $$\begin{array}{c} \int_{0}^{\infty }\xi^{\left|m\right|}e^{-\xi }F^{2}(-n_{\rho },|m|+1,\xi ){\left(\frac{\left|m\right|}{\xi }+2\frac{F^{\prime }(-n_{\rho },|m|+1,\xi )}{F(-n_{\rho },|m|+1,\xi )}-2\frac{L_{n_{\rho }}^{\prime }\left(\xi \right)}{L_{n_{\rho }}\left(\xi \right)}\right)}^{2}d\xi , \end{array}$$

which is a challenge both analytically and numerically. In a specific case of $n_{\rho }=0$, i.e., $\eta_{0,0}\left(\xi \right)=e^{-\xi }$, we provide the results in Appendix A, where we find that the curves are qualitatively similar to Figure 1 and Figure 2.

While the amount of Fisher information of the Landau states is independent of the imposed magnetic field in terms of an independent variable $\xi =\rho^{2}\left|e\right|H/2\hbar$, the electron energy levels, that is, the Landau levels are inevitably dependent on it and they are provided as [1,3,22]

(15) $$\begin{array}{c} E=\hbar \omega \left(n_{\rho }+\frac{\left(|m|+m+1\right)}{2}\right)+\frac{p_{z}^{2}}{2M}, \end{array}$$

where ω is the angular frequency of the circular motion in a plane perpendicular to the uniform magnetic field (i.e., cyclotron frequency |e|H/Mc) and $p_{z}$ is the momentum component along the field. This formula indicates that the sign of the azimuthal quantum number matters for the energy of oscillation. However, the wavefunctions corresponding to the Landau states have the index as absolute values. Thus, Fisher information associated with the states degenerates. In other words, apart from the split of the Landau level due to the electron spin, the states with m>0 have higher perpendicular energy by mℏω than the corresponding m<0 states, while the amount of Fisher information is the same. This asymmetry inherent in the Landau states was also emphasized in the experiment of electron rotations in a magnetic field, where the electrons are found to rotate with three different angular velocities depending on the sign of *m* or m=0 [21].

## Figures and Tables

**Figure A1:**
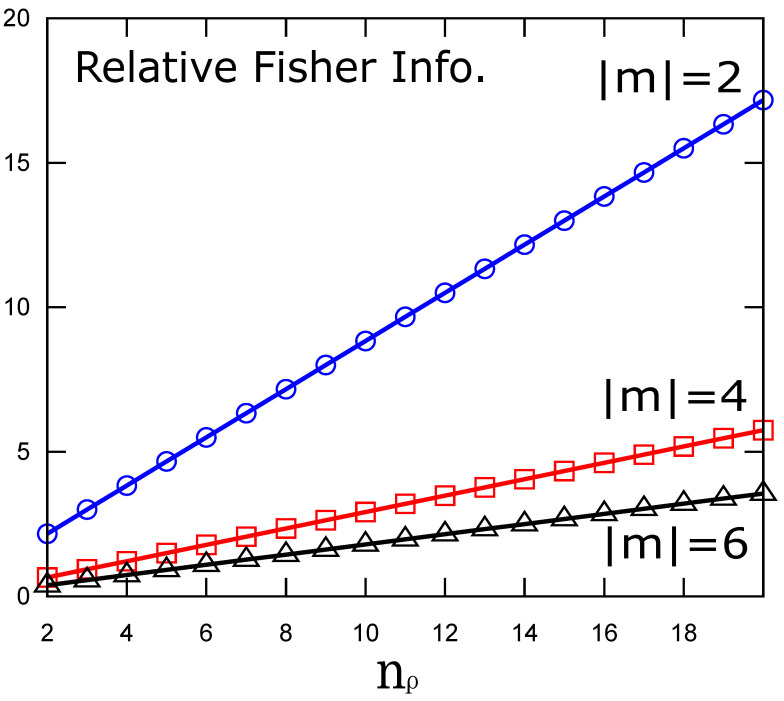
Relative Fisher information of the Landau states as a function of the radial quantum number $n_{\rho }$ for three different values of |m|.

**Figure A2:**
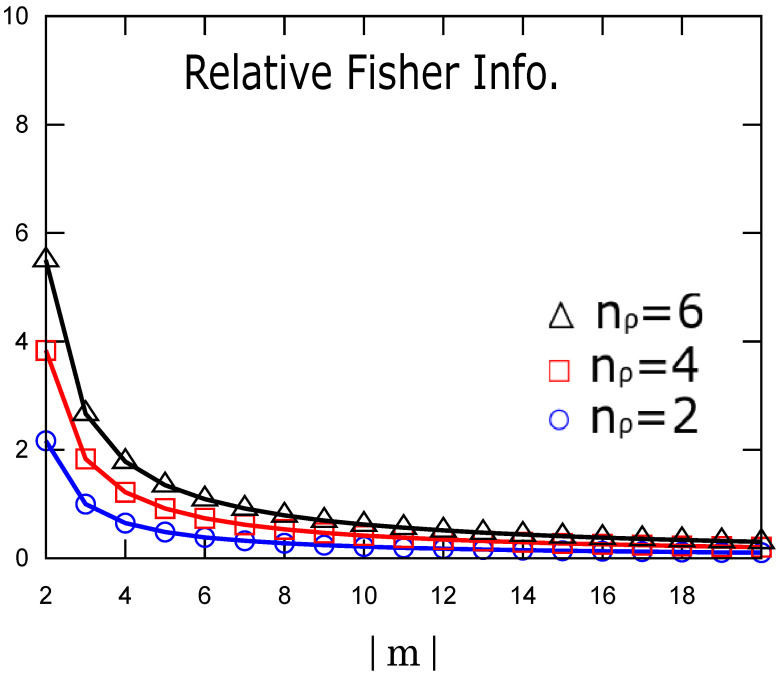
Relative Fisher information of the Landau states as a function of the azimuthal quantum number |m| for three different values of $n_{\rho }$.

## Appendix A. Relative Fisher Information of Landau States with $\left(n_{\rho },|m|\right)$ with Respect to the Lowest State (0,0)

We calculate the relative Fisher information against the lowest Landau state (0,0), i.e., $\mathrm{RFI}(\eta_{n_{\rho },m}\left(\xi \right):\eta_{0,0}\left(\xi \right))$. This quantity provides how much the probability density of the Landau states deviates from the exponentially decreasing probability density $\eta_{0,0}\left(\xi \right)=e^{-\xi }$. In Equation (14), we note $L_{0}\left(\xi \right)=1$. Then, expanding the integrand and using Equation (11) combined with the relation ${\left[L_{n}^{\left(\alpha \right)}\left(\xi \right)\right]}^{\prime }=-L_{n-1}^{\left(\alpha +1\right)}\left(\xi \right)$, we can express it as

(A1) $$\begin{array}{c} \mathrm{RFI}\left(\eta_{n_{\rho },m}\left(\xi \right):\eta_{0,0}\left(\xi \right)\right)=\frac{n_{\rho }!}{(n_{\rho }+|m|)!}\left(I_{1}+I_{2}+I_{3}\right), \end{array}$$

where we have put the integrals, respectively, as

(A2) $$\begin{array}{ccc} I_{1} & = & {\left|m\right|}^{2}\int_{0}^{\infty }\xi^{|m|-2}e^{-\xi }{\left[L_{n_{\rho }}^{\left(|m|\right)}\left(\xi \right)\right]}^{2}d\xi \\ I_{2} & = & -4|m|\int_{0}^{\infty }\xi^{|m|-1}e^{-\xi }L_{n_{\rho }}^{\left(|m|\right)}\left(\xi \right)L_{n_{\rho }-1}^{\left(|m|+1\right)}\left(\xi \right)d\xi \\ I_{3} & = & 4\int_{0}^{\infty }\xi^{\left|m\right|}e^{-\xi }{\left[L_{n_{\rho }-1}^{\left(|m|+1\right)}\left(\xi \right)\right]}^{2}d\xi . \end{array}$$

We evaluate these integrals below. Note that similar integrals also appear in calculating relative Fisher information for the *D*-dimensional isotropic quantum oscillator [28]. There would be several ways to obtain these integrals. Here, we use an integral formula involving the product of two Laguerre polynomials with different integer degrees (*p* and *q*) and orders (α and β), which was evaluated by Mavromatis [32]:

(A3) ∫0∞xμe−xLp(α)(x)Lq(β)(x)dx=p+αpq+β−μ−1qΓ(μ+1)×3F2(−p,μ+1,μ−β+1;α+1,μ−β−q+1;1),

where the power exponent satisfies Re(μ)>−1 and ab denotes the binomial coefficient. As usual, ${}_{3}F_{2}$ is a generalized hypergeometric series with three numerator and two denominator parameters.

Integral $I_{1}$:

Putting μ=|m|−2, α=β=|m|, and $p=q=n_{\rho }$ in Equation (A3), we have

(A4) $$\begin{array}{c} I_{1}=\frac{(n_{\rho }+|m|)!}{|m|!n_{\rho }!}\left(n_{\rho }+1\right)\Gamma {\left(|m|-1\right)}_{3}F_{2}(-n_{\rho },|m|-1,-1;|m|+1,-n_{\rho }-1;1). \end{array}$$

where |m|≠1.

Integral $I_{2}$:

Putting μ=|m|−1, α=|m|, β=|m|+1, $p=n_{\rho }$, and $q=n_{\rho }-1$ in Equation (A3), we have

(A5) $$\begin{array}{c} I_{2}=\frac{(n_{\rho }+|m|)!}{|m|!n_{\rho }!}n_{\rho }\Gamma {\left(|m|\right)}_{2}F_{1}\left(|m|,-1;|m|+1;1\right), \end{array}$$

where ${}_{2}F_{1}$ is the Gauss’ hypergeometric function. By using the formula for the particular value of unity (p. 1017 in Ref. [31]),

(A6) $$\begin{array}{c} {}_{2}F_{1}\left(\alpha ,\beta ,\gamma ;1\right)=\frac{\Gamma (\gamma )\Gamma (\gamma -\alpha -\beta )}{\Gamma (\gamma -\alpha )\Gamma (\gamma -\beta )}, \end{array}$$

we find that this factor reduces to ${\left(|m|+1\right)}^{-1}$.

Integral $I_{3}$:

Putting μ=|m|, α=β=|m|+1, and $p=q=n_{\rho }-1$ in Equation (A3), we have

(A7) $$\begin{array}{ccc} I_{3} & = & \frac{(n_{\rho }+|m|)!}{\left(|m|+1\right)!\left(n_{\rho }-1\right)!}\Gamma {\left(|m|+1\right)}_{3}F_{2}(-n_{\rho }+1,|m|+1,0;|m|+2,-n_{\rho }+1;1)\\ & = & \frac{(n_{\rho }+|m|)!}{|m|!n_{\rho }!}\frac{|m|!n_{\rho }}{|m|+1}. \end{array}$$

where we have used that the value of ${}_{3}F_{2}$ becomes 1 because the one of the numerators vanishes. Combining these results Equations (A4), (A5), and (A7), we obtain the value of $\mathrm{RFI}(\eta_{n_{\rho },m}\left(\xi \right):\eta_{0,0}\left(\xi \right))$. Figure A1 and Figure A2 show relative Fisher information Equation (A1) as a function of $n_{\rho }$ and |m|, respectively. In contrast to the constant slope of 4 derived in Equation (13) when one sets the reference density as $\eta_{0,m}$, the linearity depends on both quantum numbers. This dependency is remarkably analogous to the case of the *D*-dimensional isotropic quantum oscillator, in which the relative Fisher information of the radial wavefunction depends on both the principal quantum number *n* and the orbital quantum number *l* [28]. We observe in Figure A1 that for a fixed $n_{\rho }$, the Landau states with a higher azimuthal quantum number get closer to the exponentially decreasing density. In contrast, the information monotonically decreases as |m| increases as shown in Figure A2 and we also find that for a fixed |m|, the states with a lower $n_{\rho }$ approach the density of the lowest Landau state.
